# Microstructure and Mechanical Properties of TiB_2_/AlSi7Mg0.6 Composites Fabricated by Wire and Arc Additive Manufacturing Based on Cold Metal Transfer (WAAM-CMT)

**DOI:** 10.3390/ma15072440

**Published:** 2022-03-25

**Authors:** Qingfeng Yang, Cunjuan Xia, Haowei Wang, Mingyang Zhou, Shixin Gao, Bingjin Li, Shichao Liu

**Affiliations:** 1 Science and Technology on Reactor System Design Technology Laboratory, Nuclear Power Institute of China, Chengdu 610213, China; 18328548365@163.com (Q.Y.); 18782951516@163.com (M.Z.); gsx5@163.com (S.G.); bingjin_li@163.com (B.L.); hit_lsc@163.com (S.L.); 2 The State Key Laboratory of Metal Matrix Composites, Shanghai Jiao Tong University, Shanghai 200240, China; hwwang@sjtu.edu.cn

**Keywords:** WAAM-CMT, TiB_2_/AlSi7Mg0.6 samples, microstructure, mechanical properties

## Abstract

Wire and arc additive manufacturing based on cold metal transfer (WAAM-CMT), as a kind of clean and advanced technology, has been widely researched recently. It was analyzed in detail for the microstructure and mechanical properties of WAAM-CMT printed TiB_2_/AlSi7Mg0.6 samples fore-and-aft heat treatment in this study. Compared with the grain size of casted AlSi7Mg0.6 samples (252 μm), the grain size of WAAM-CMT printed AlSi7Mg0.6 samples (53.4 μm) was refined, showing that WAAM-CMT process could result in significant grain refinement. Besides, the grain size of WAAM-CMT printed TiB_2_/AlSi7Mg0.6 samples was about 35 μm, revealing that the addition of TiB_2_ particles played a role in grain refinement. Nevertheless, the grain size distribution was not uniform, showing a mixture of fine grain and coarse grain, and the mechanical properties were anisotropic of the as-printed samples. This study shows that T6 heat treatment is an efficient way to improve the nonuniform microstructure and eliminate the anisotropy in mechanical properties.

## 1. Introduction

Aluminium alloys, with high strength, low density, and good corrosion resistance, have been widely used in many industries [1,2,3]. Additive manufacturing (AM), as a kind of clean and advanced technology, has been popular recently, which fabricates the samples layer-by-layer according to 3D models. This technology could avoid material waste and reduce technological processes, which enable the formation of any complex components theoretically [4,5,6,7]. Additive manufacturing could be classified as laser additive manufacturing [8], electron beam additive manufacturing [9], and arc additive manufacturing according to the heat source [10]. Wire and arc additive manufacturing based on cold metal transfer (WAAM-CMT) technology is very appropriate for the formation of aluminium alloys because of its merits of low heat input and high process speed [11,12,13,14].

The forming characteristics of WAAM and the microstructure and properties of the printed parts have become a hot topic in the field of additive manufacturing. Blanka et al. [15] studied the microstructure and residual stress in Ti-6Al-4V AM components produced by Wire + Arc Additive Manufacturing (WAAM) and by laser cladding process (CLAD). Su et al. [16] studied the microstructure and properties of Al-Mg alloys fabricated by WAAM under different heat inputs, finding grain size varying in the range of 42.9–88.7 μm in the inner-layer region and 37.7–77.6 μm in the inter-layer region. Patel et al. [17] focused on the effects of different forming process parameters and heat-treatment regimens on the microstructure and properties of formed parts, holding a view that it was necessary for the printed parts to process heat treatment in order to meet the requirements. Jiang et al. [18] researched the effect of process parameters, such as current, scanning speed, and so on, on the forming quality of WAAM-CMT printed 5356 aluminum alloy, finding there was a difference in tensile strength in different directions. Bai et al. [19] studied the microstructure of 4043 aluminum alloy printed by WAAM, which consisted of texture and elements segregation. Moreover, Huang et al. [20] and Sun et al. [21] both found similar phenomena in printed 5A06 aluminum alloy and Al-6.3Cu aluminum alloy respectively. From the studies mentioned above, it might be a common phenomenon that there were anisotropy and non-uniform microstructure in printed samples, which was detrimental to the comprehensive performance of samples [15,22,23]. It is a key research point to enhance the microstructure and mechanical properties of printed samples. Besides, there is little research on parts fabricated with wire and arc additive manufacturing by feeding composite wire [24,25,26].

In this study, the microstructure and mechanical properties of TiB_2_/AlSi7Mg0.6 composites printed by WAAM-CMT technology were found. In addition, the effect of heat treatment on WAAM-CMT printed TiB_2_/AlSi7Mg0.6 composites was analyzed and the mechanisms of improving microstructure and mechanical properties were discussed correspondingly.

## 2. Materials and Methods

**Figure 1:**
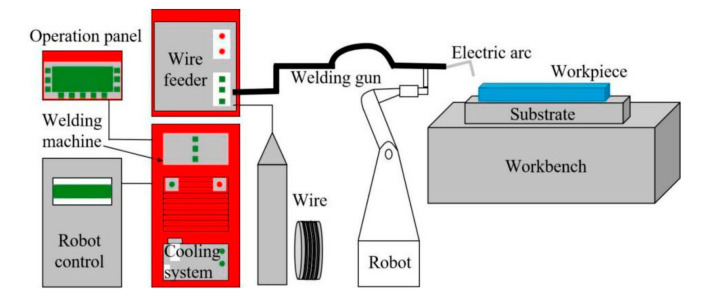
The schematic diagram of WAAM-CMT equipment.

In this study, the in-situ authigenic TiB_2_/AlSi7Mg0.6 ingot was prepared first through the mixed-salt method, then the ingot was transformed into wire used in WAAM by a series of processing techniques, such as extrusion, drawing, and so on. The WAAM-CMT equipment (NanJing Enigma Automation CO., LTD, Nanjing, China) mainly consists of a welding robot, wire feeder system, cooling system, robot control system, and workbench, as shown in Figure 1, where Φ 1.6mm TiB_2_/AlSi7Mg0.6 composite wire and 4043 aluminum alloy as the substrate were used. During the whole additive manufacturing process, pure argon gas, as protective gas, was used to avoid the oxidation of materials.

**Figure 2:**
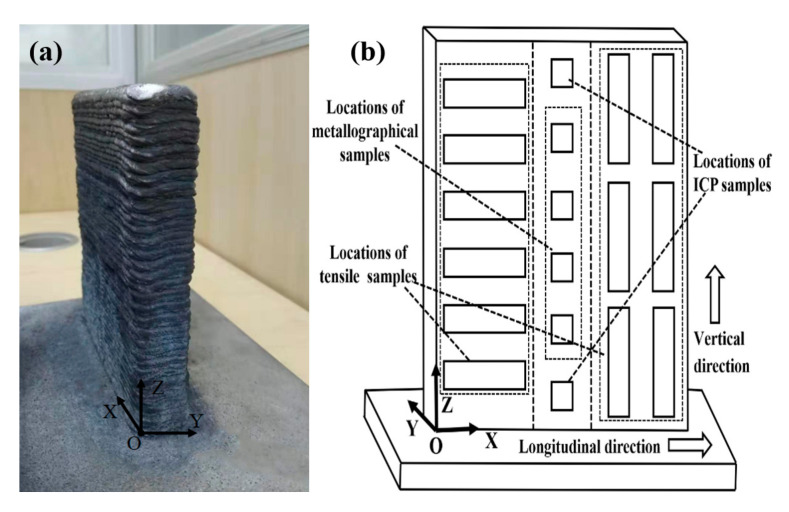
(**a**) WAAM-CMT printed TiB_2_/AlSi7Mg0.6 sample; (**b**) extraction locations of specimens and definition of X, Y, and Z directions.

During the process of WAAM-CMT, welding voltage, welding current, wire feed speed, and travel speed was 15.1V, 145A, 7.3 m/min, and 12 mm/s respectively. The WAAM-CMT printed TiB_2_/AlSi7Mg0.6 sample is shown in Figure 2a. The X direction was defined as along the travel direction, the Y direction was defined as perpendicular to the travel direction, and the Z direction was defined as along the height direction (Figure 2b). Additionally, the WAAM-CMT printed TiB_2_/AlSi7Mg0.6 sample was processed by T6 heat treatment (solid solution 535 ^◦^C/1h + quenching + artificial aging 120 ^◦^C/1h + 165 ^◦^C/8h).

In order to analyze the homogeneity of chemical composition, specimens were extracted from different locations, using ICAP6300 inductively coupled plasma optical, and the result is shown in Table 1.

Furthermore, optical microscopy (OM) (Carl Zeiss (Shanghai) Management Co., Ltd, Shanghai, China.) and scanning electron microscope (SEM) (TESCAN China, Shanghai, China) were selected to study the microstructure of the specimens. For elements analysis and statistical analysis of grain size, energy disperse spectroscopy (EDS) (TESCAN China, Shanghai, China) and electron backscattered diffraction (EBSD) (TESCAN China, Shanghai, China) equipment were used respectively. In addition, tensile tests at ambient temperature were performed with the strain rate 1 × 10^−4^ s^−1^.

## 3. Results and Discussion

### 3.1. Microstructure Evolution

**Figure 3:**
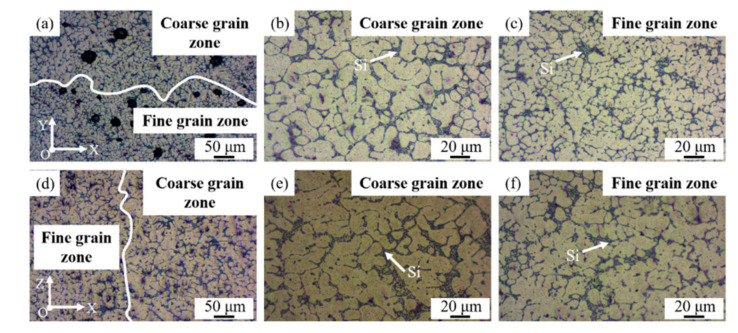
Metallographic morphology of the WAAM-CMT-printed TiB_2_/AlSi7Mg0.6 sample in face XOY and XOZ: (**a**) face XOY; (**b**) coarse grain zone in (**a**); (**c**) fine grain zone in (**a**); (**d**) face XOZ; (**e**) coarse grain zone in (**d**); (**f**) fine grain zone in (**d**).

The microstructures of the as-deposited TiB_2_/AlSi7Mg0.6 composites in face XOY and face XOZ, composed of fine grain and coarse grain, were similar (Figure 3). Besides, it seemed that the size of dendrites in face XOZ was larger than that in face XOY. Additionally, dendritic α-Al phase and strip-like Si phase occurred in the metallographic morphology of the WAAM-CMT-printed TiB_2_/AlSi7Mg0.6 sample in two faces.

The appearance of fine grain and coarse grain was connected with the WAAM-CMT process. During the formation process, dendritic crystals grew along the direction of the temperature gradient, which was along the height direction. In addition, the cooling rate at the bottom of the molten pool was very high, forming fine crystals. There were larger dendrites in the middle of the molten pool because of a higher temperature than that at the bottom. While, fine dendrites existed at the top of the molten pool on account of high heat dissipation. Furthermore, when forming at the upper layer, solidified materials at the lower layer would melt again, resulting in the growth of crystals in this region [27,28,29].

**Figure 4:**
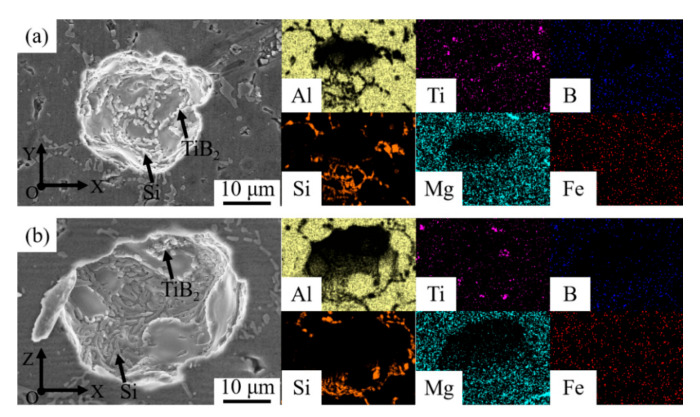
Stomata morphology of the WAAM-CMT-printed TiB_2_/AlSi7Mg0.6 sample in face XOY and XOZ: (**a**) face XOY; (**b**) face XOZ.

Figure 3a shows that there were pores with different sizes along the division of the fine grain zone and coarse grain zone, the larger diameter of which was up to about 40 µm. Some scholars hold a view that it is easier for pores to form at the solidification interface of dendrites because of the irregular shape of the solid-liquid interface, reducing the nucleation work of pores formation [30,31]. Shivkumar et al. [32] proposed that pores formed based on the dendrite arm and the shorter the distance between secondary branches was, the smaller the nucleation radius of pores was. The above was the reason why there were pores along the division of fine grain zone and coarse grain zone. The pore morphology of the WAAM-CMT formed samples was observed, as shown in Figure 4.

It could be seen that there were mainly Si particles and a small amount of segregated TiB_2_ particles on the inner wall of the pores. Moreover, the distribution of Si on the inner wall of the pores was obviously denser than that on the Al matrix.

**Figure 5:**
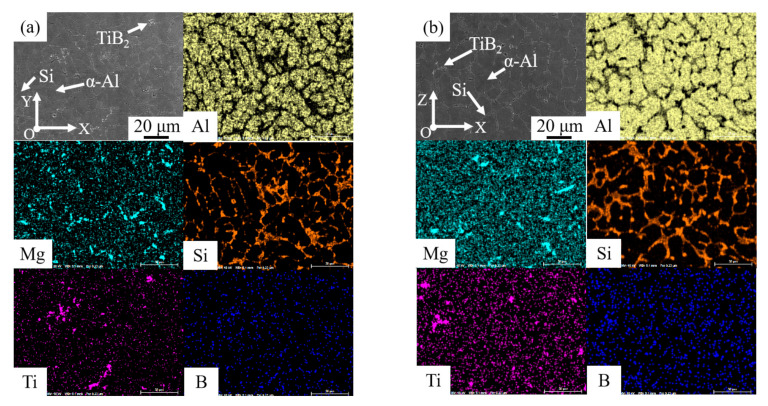
Microstructure of the WAAM-CMT-printed TiB_2_/AlSi7Mg0.6 sample in face XOY (**a**) and XOZ (**b**).

For farther analysis of the microstructure of printed TiB_2_/AlSi7Mg0.6 composites in two faces (Figure 5), it could be seen that most parts of Si particles were distributed along the α-Al grain boundary and some TiB_2_ particles segregated at the grain boundary. The size of Si particles was about 3 to 6 µm. Furthermore, there was elements segregation at the grain boundary in printed TiB_2_/AlSi7Mg0.6 samples, which was a result of the high cooling velocity of the WAAM-CMT printing process. When the cooling velocity of molten pool was high, there would be less time for elements to diffuse sufficiently, finally causing the elements segregation [22,29].

**Figure 6:**
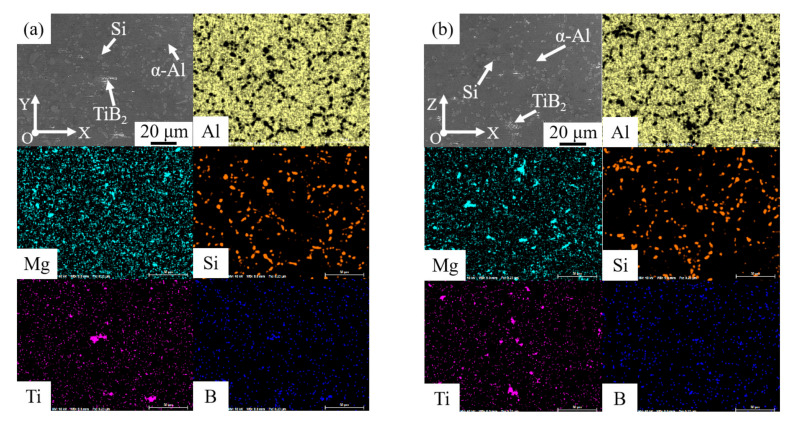
Microstructure of WAAM-CMT printed TiB_2_/AlSi7Mg0.6 composites after T6 heat treatment in face XOY (**a**) and XOZ (**b**).

After T6 heat treatment, the division between the coarse grain zone and fine grain zone seemed to disappear. Furthermore, it was evident that the elements distribution in Figure 6 was more uniform than that in Figure 5, because the elements had enough time and energy to diffuse during the process of T6 heat treatment. In addition, the shape of Si particles was changed to block from stripe, with a size of about 2–6 µm, which was also related to the element diffusion. In the microstructure of WAAM-CMT-printed TiB_2_/AlSi7Mg0.6 composites, the silicon atoms at the sharp corners had higher energies, which would migrate to the location with lower energies during the process of heat treatment, leading to the shape change of Si particles [33,34].

**Figure 7:**
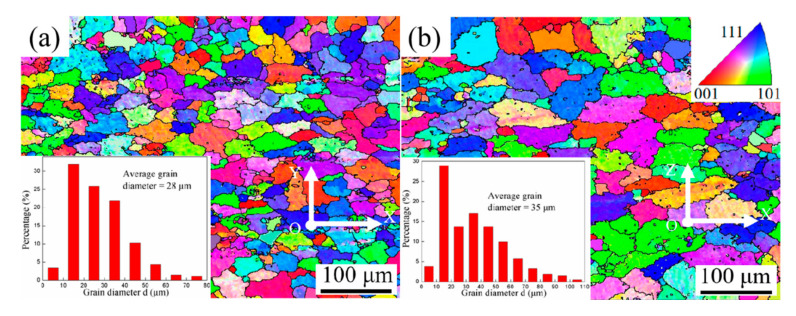

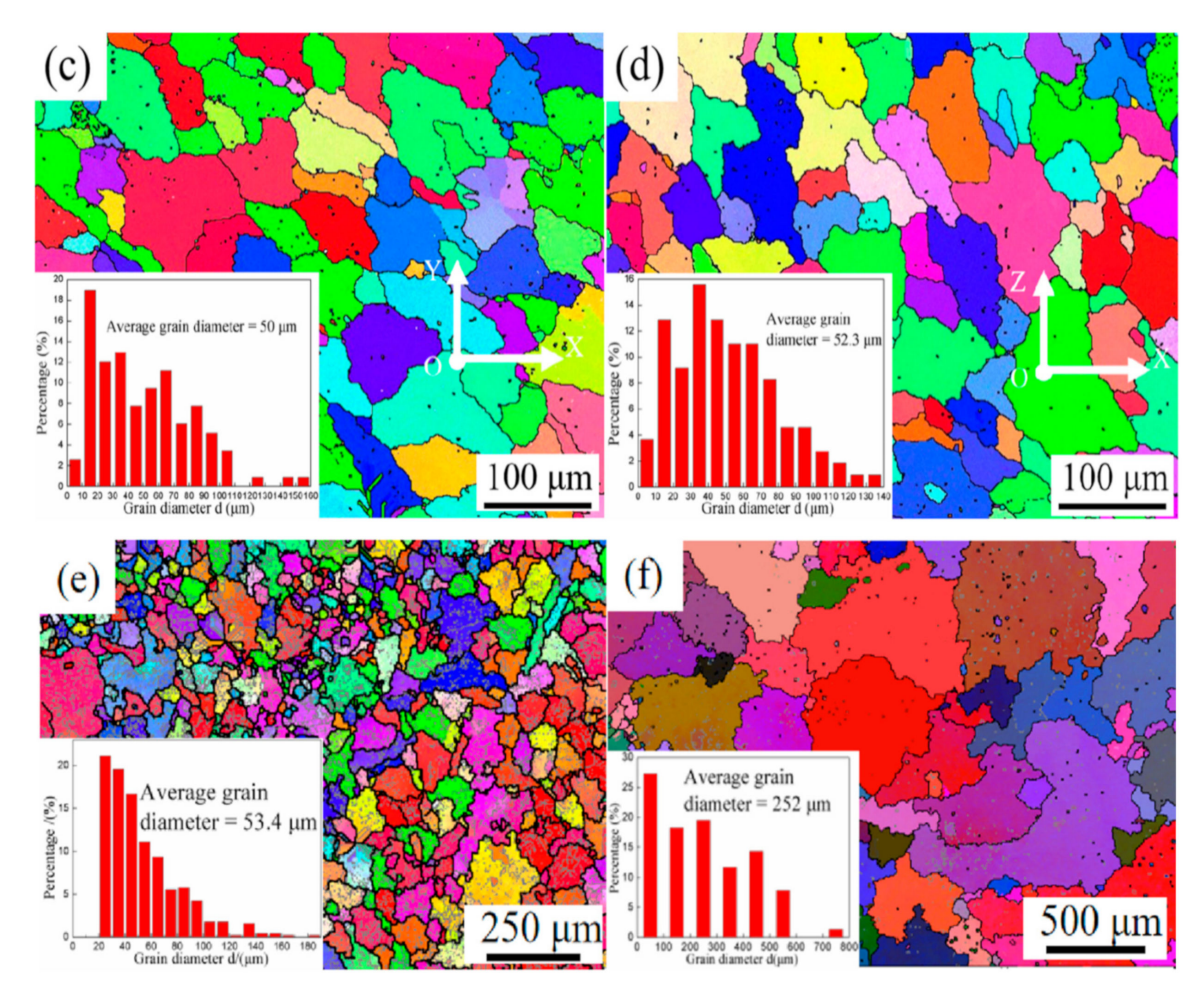
EBSD graphs and grain size diagrams of samples: (**a**) face XOY of WAAM-CMT-printed TiB_2_/AlSi7Mg0.6 composites; (**b**) face XOZ of WAAM-CMT-printed TiB_2_/AlSi7Mg0.6 composites; (**c**) face XOY of WAAM-CMT-printed TiB_2_/AlSi7Mg0.6 composites after T6 heat treatment; (**d**) face XOZ of WAAM-CMT-printed TiB_2_/AlSi7Mg0.6 composites after T6 heat treatment; (**e**) WAAM-CMT-printed AlSi7Mg0.6 alloy; (**f**) casted AlSi7Mg0.6 alloy.

According to the average grain size and grain size distribution of the printed composites in face XOY and face XOZ (Figure 7a,b), it could be seen that those were a little different in two faces and the average grain size in face XOZ was larger than that in face XOY. After T6 heat treatment, the average grain size in XOY and XOZ had an evident increase of 78.57% and 49.43% respectively. The growth of grain size after heat treatment was because of the decrease of total free energy of the whole system. During the process of grain growth, grains with small size, less than a certain critical value, disappeared gradually, while the grains with large size grew further [35].

Besides, the average grain size of casted AlSi7Mg0.6 alloys was much larger than that of printed AlSi7Mg0.6 alloys, despite that of printed TiB_2_/AlSi7Mg0.6 composites, which indicated that WAAM-CMT printing process and the addition of TiB_2_ particles both played roles of refining grain. As the mass of the addition of TiB_2_ particles in composites was small, only about 0.5 wt%, the effect was limited for TiB_2_ particles to inhibit grain growth after T6 heat treatment.

The reason for grain refinement is explained as follows. Firstly, TiB_2_ particles was favorable to the increase of nucleation rate as there was a co-lattice relationship between TiB_2_ lattice and α-Al lattice [28]. Secondly, the cooling rate of the WAAM-CMT printing process, about 245 K/s, was much higher than that of the casting process [36]. The higher the cooling rate was, the higher the nucleation rate was, leading to eventual grain refinement. Finally, arc stir in the molten pool also exerted a positive effect on grain refinement. Even some scholars held a view that the arc stir effect of the CMT mode was most notable among other arc modes [37,38].

### 3.2. Mechanical Properties Analysis

For the purpose of researching the mechanical properties of TiB_2_/AlSi7Mg0.6 composites in different directions, samples were extracted from X and Z directions (Figure 2).

**Figure 8:**
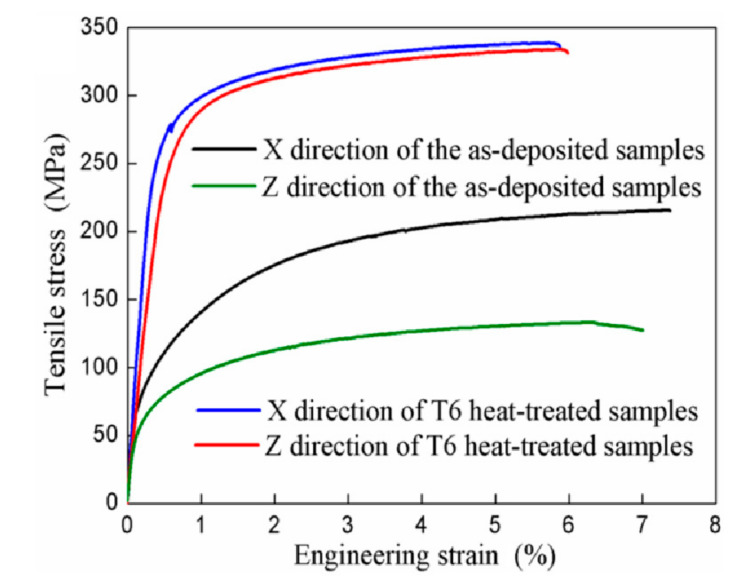
Tensile test of TiB_2_/AlSi7Mg0.6 composites.

Tensile test results (Figure 8 and Table 2) showed that the yield strength (R_p0.2_) and ultimate tensile strength (R_m_) in the X direction were obvious higher than those in the Z direction, because the average grain size in face XOZ of printed composites was larger than that in face XOY. That was to say, there was anisotropy in mechanical properties of WAAM-CMT-printed TiB_2_/AlSi7Mg0.6 composites, which was related to the difference in microstructure in different directions. During the tensile test, dislocations would move along the force direction in general, and the movement of dislocations would be hindered because of the existence of grain boundary. The finer the grain size was, the higher the strength of sample was [35].

After T6 heat treatment, the value of yield strength, ultimate tensile strength, and elongation (A) in two directions were the same on the whole, indicating that T6 heat treatment was an effective method to eliminate the anisotropy of WAAM-CMT-printed TiB_2_/AlSi7Mg0.6 composites. Besides, the value of yield strength and ultimate tensile strength in two directions both clearly increased after heat treatment, while there was a decrease of elongation in two directions.

In general, the yield strength of samples would decrease if the grain sizes of samples increased [39]. However, even though the grain sizes of printed composites increased after heat treatment, there was an increase of yield strength, indicating that precipitation strengthening and the solid solution of Si particles led to the increase of yield strength. For one thing, the following was the general precipitation sequence in Al-Si-Mg alloy after heat treatment, super-saturated solid solution → GP zones → β” → β’ → β, among which the β” phase, as generally assumed, played the most important role in precipitate strengthening [40]. For another, TiB_2_ particles with micro or nano size could have a similar effect of precipitate strengthening. Besides, parts of Si particles, with high elasticity modulus, would dissolve in the α-Al matrix, increasing the yield strength as well [41]. It is proved that appropriate heat treatment is beneficial to improve the comprehensive mechanical properties of the parts made by WAAM-CMT.

**Figure 9:**
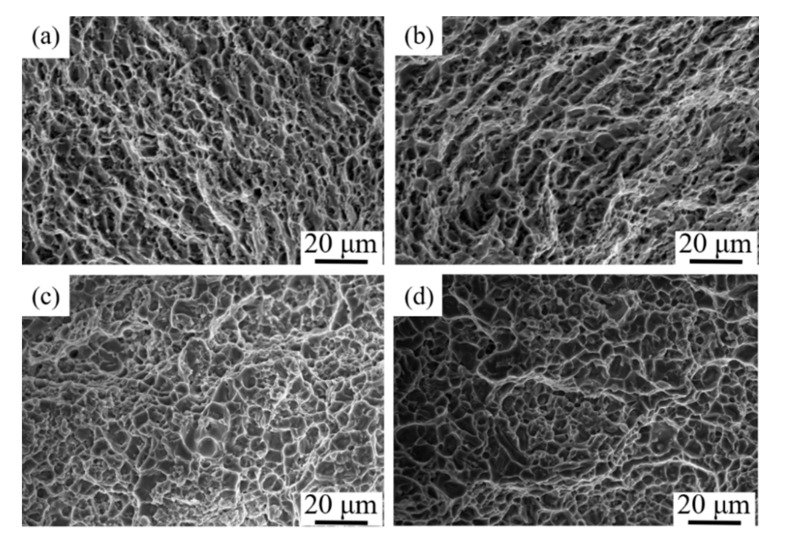
Fracture morphology of tensile-tested specimens: (**a**) X direction in the as-deposited samples; (**b**) Z direction in the as-deposited samples; (**c**) X direction in the T6 heat-treated samples; (**d**) Z direction in the T6 heat-treated samples.

SEM graphs of the fracture of the samples are shown in Figure 9a–d. It can be seen that there were dimples in the fracture morphologies of the as-deposited and T6 heat-treated samples. The dimples suggest that most of the failure is because of ductile fracture.

## 4. Conclusions

The microstructure and mechanical properties of WAAM-CMT-printed TiB_2_/AlSi7Mg0.6 composites were analyzed systematically, obtaining the following conclusions.

1. There were fine grain and coarse grain zones in the microstructure of printed TiB_2_/AlSi7Mg0.6 composites, and it seemed that T6 heat treatment had an advantage of eliminating the division between fine grain and coarse grain zones.
2. The grain size of the printed AlSi7Mg0.6 alloy was much smaller than that of the casted AlSi7Mg0.6 alloy as the result of the high cooling rate of WAAM-CMT printing process. The grain size of printed TiB_2_/AlSi7Mg0.6 samples was smaller than that of the printed AlSi7Mg0.6 alloy, indicating that TiB_2_ particles also played a role in refining grain.
3. After T6 heat treatment, there was an obvious increase of yield strength and ultimate tensile strength of the composites in X and Z directions, mainly on account of the precipitation strengthening and solid solution of Si particles.

## Figures and Tables

**Table 1:** Chemical composition of TiB_2_/AlSi7Mg0.6 wire and samples (mass fraction, wt%).

Element	Si	Mg	Ti	B	Al
Wire	6.79	0.67	1.22	0.58	Bal.
Upper location of the sample	6.72	0.59	0.45	0.21	Bal.
Lower location of the sample	6.75	0.62	0.53	0.23	Bal.

**Table 2:** Mechanical properties of TiB_2_/AlSi7Mg0.6 composites fore-and-aft T6 heat treatment.

State	R_p0.2_ (MPa)	R_m_ (MPa)	A (%)
Printed samples	103 ± 7.26 (X)	216 ± 10.87 (X)	7 ± 0.89 (X)
	83 ± 6.54 (Z)	134 ± 9.99 (Z)	7 ± 0.77 (Z)
T6 heat-treated samples	270 ± 7.62 (X)	340 ± 8.18 (X)	6 ± 0.63 (X)
	266 ± 7.43 (Z)	335 ± 8.75 (Z)	6 ± 0.58 (Z)

## Data Availability

Not applicable.
